# Profiling of the Mammalian Mitotic Spindle Proteome Reveals an ER Protein, OSTD-1, as Being Necessary for Cell Division and ER Morphology

**DOI:** 10.1371/journal.pone.0077051

**Published:** 2013-10-10

**Authors:** Mary Kate Bonner, Bo Hwa Han, Ahna Skop

**Affiliations:** Laboratory of Genetics and Medical Genetics, University of Wisconsin-Madison, Madison, Wisconsin, United States of America; University of North Carolina, United States of America

## Abstract

Cell division is important for many cellular processes including cell growth, reproduction, wound healing and stem cell renewal. Failures in cell division can often lead to tumors and birth defects. To identify factors necessary for this process, we implemented a comparative profiling strategy of the published mitotic spindle proteome from our laboratory. Of the candidate mammalian proteins, we determined that 77% had orthologs in *Caenorhabditis elegans* and 18% were associated with human disease. Of the *C. elegans* candidates (n=146), we determined that 34 genes functioned in embryonic development and 56% of these were predicted to be membrane trafficking proteins. A secondary, visual screen to detect distinct defects in cell division revealed 21 genes that were necessary for cytokinesis. One of these candidates, OSTD-1, an ER resident protein, was further characterized due to the aberrant cleavage furrow placement and failures in division. We determined that OSTD-1 plays a role in maintaining the dynamic morphology of the ER during the cell cycle. In addition, 65% of all *ostd-1* RNAi-treated embryos failed to correctly position cleavage furrows, suggesting that proper ER morphology plays a necessary function during animal cell division.

## Introduction

Cell division is a fundamental process that functions in cell growth, development, and stem cell renewal. Errors in cell division can often lead to tumors, birth defects and failure to regenerate stem cells [1-5]. Cell division is mediated by a complex machine called the mitotic spindle which is necessary for chromosome segregation and cell separation [6-11]. The structure of the mitotic spindle is comprised of spindle microtubules, centrosomes, and chromosomes [12]. The proteins associated with centrosomes and mitotic microtubules have a wide range of functions that regulate the strength and dynamic nature of the spindle [13-22]. Microtubule binding proteins lend strength and stability to the spindle structure [23,24], while motor proteins and kinases regulate the movement of the mitotic spindle, trafficking of factors, and the progression of cell division [17,21-24]. Spindle-based mRNAs also play an important role in localized translation of factors important for spindle function [25,26]. Lastly, membrane proteins play a necessary role in spindle dynamics and cell division [15,20,27-30]. Despite all of the knowledge about this complex machine, many of the factors and signaling pathways that mediate mitotic events during cell division remain unclear.

In recent years, a wealth of information about the protein composition of the mitotic spindle has been obtained through proteomics, genomics and bioinformatics assays on mitotic structures. Proteomic studies of microtubules and mitotic structures, in particular, have identified numerous proteins associated with the mitotic spindle [13-17,19-22]. In *Drosophila, Schizosaccharomyces pombe*, and *Caenorhabditis elegans*, numerous factors necessary for cytokinesis have been identified through the combination of forward genetics, RNAi approaches, and chemical genetics screens [15,31-40]. Transcriptomic studies of mitotic microtubules have revealed that RNAs associated with the mitotic spindle are likely important for localized protein synthesis during mitosis [25,26]. Despite the identification of numerous factors, the functions of many of these proteins and RNAs are unclear. However, directed RNAi and chemical genomic screens have been more fruitful in investigating the functions of smaller groups of genes in a focused context. Here, the incorporation of *in vivo* video microscopy to record phenotypes in detail has impacted our knowledge of spindle function immensely [15,34,35]. With the addition of bioinformatics*, in silico* screens combined with single gene analyses have revealed, “hidden spindle hubs” important for global spindle function [41]. Overall, the examination of smaller groups of mitotic genes has allowed for detailed characterization of subtle phenotypic variations with fewer errors. 

To identify necessary cell division factors from the mammalian mitotic spindle proteome recently published by our lab [20], we developed a comparative profiling strategy and visual screen of corresponding orthologs in *C. elegans*. Proteins from the CHO cell spindle proteome were first filtered by GO terms (http://geneontology.org) to identify a group of candidate genes. We then identified the corresponding *C. elegans* orthologs. To determine if the *C. elegans* orthologs were important for embryonic development, we performed an RNAi screen for embryonic lethality and profiled this information from previous genome-wide screens in *C. elegans* [15,34,35]. Next, we performed a visual GFP-based RNAi screen to determine which genes function in cell division. Initial characterization of one candidate, OSTD-1, was performed.

## Results

### Conserved mitotic spindle genes have links to human disease

To identify which mitotic spindle candidate genes play a role in human disease, we investigated the Online Mendelian Inheritance in Man (OMIM) database [http:://omim.org/]. Of all of our candidates, 18.1% (n=58/320) were associated with human disease (labeled with an asterisk in the Table S1). Several of the genes were linked to multiple types of cancer, deafness and neurological disorders. Exploring phenotypic similarities and differences between *C. elegans* embryos and humans will likely generate insights into cellular mechanisms underlying diseases in the future. 

### Identifying conserved mammalian mitotic spindle genes in *C. elegans*

To determine the corresponding *C. elegans* orthologs for our candidate mitotic spindle proteins, we used several bioinformatic approaches including GO terms, reciprocal BLAST results, identifying protein domains on Pfam and determining the known orthologs on the Treefam database. We used a multi-pronged strategy to determine orthologs (see Methods), similar to the strategy of the recently published *C. elegans* Ortholist [42]. In addition, we manually annotated each protein using UniProt, InterPro and Ensembl databases and also referred to the published literature for known orthologs. Our starting candidate protein list included proteins associated the following GO terms: actin, microtubules, membrane trafficking, proteasome and an unknown group. As GO terms can be predictive of protein function [43,44], we prioritized the membrane-cytoskeletal groups. We also included the proteasome group in our RNAi screen, which were identified in the midbody proteome but not included in the original midbody RNAi screen [15]. Proteins without GO terms or that had not been previously annotated for localization, labeled as unknown, were also added to this list [20]. Overall, 77% of the published mammalian mitotic spindle candidates (n=320) had *C. elegans* orthologs, suggesting that the mitotic spindle protein composition is well conserved. Next, we removed *C. elegans* genes with previously characterized roles in mitosis and cytokinesis, leaving us with 146 genes that we assayed in an embryonic lethality screen (see Table S1).

### Screen for embryonic lethality in *C. elegans*

 To determine which genes are important for mitotic events, we first identified genes that were important for embryonic development. To do this, *C. elegans* hermaphrodites were grown on RNAi feeding bacteria for 48 hours and then assayed for embryonic lethality. After 48 hours of feeding on RNAi bacteria, 34 genes (23.3% of the total genes) each resulted in having at least 10% embryonic lethality. To determine how effective our RNAi assay was in detecting embryonic lethality phenotypes, we explored previous RNAi data from published genome-wide screens [34,36,38,40]. Work from the Piano and Hyman labs had suggested that highly conserved genes are more likely to be necessary for cellular processes and embryonic development [37,39]. In our screen, we assayed 146 conserved *C. elegans* orthologs from our *Cricetulus griseus* (hamster; CHO cells) spindle proteome candidates. After our feeding RNAi screen, we observed a higher incidence (23.3%) of genes necessary for embryonic survival than genome-wide RNAi screens (8-14%): Maeda et al., 2001 (n=347/2479), Kamath et al., 2003 (n=1722/16707), Simmer et al., 2003 (n=2079/16401), Sonnichsen et al., 2005 (n=1668/19075) [34,36,38,40]. Feeding RNAi is also not as potent as injection [34,45], so the percentages of genes necessary for embryonic development may grow if the gene list is screened using RNAi injection techniques. We suggest that prior determination of conserved genes (between *C. griseus* (CHO) and *C. elegans*) resulted in a higher incidence of embryonic lethal genes. Given how fundamental the process of cell division is in all organisms, our results also suggest that there is a core set of conserved spindle-associated genes necessary for cell division. Our results support previous research that well conserved genes are more likely to have indispensable roles in embryonic development [37,39]. 

Work from the Piano lab demonstrated that a higher penetrance of a particular phenotype often correlates with reproducibility in *C. elegans* RNAi screens [39]. To look at these relationships, we analyzed the percentage of embryonic lethality among our candidate genes. We determined that 79% of our genes (27/34) had high levels of embryonic lethality (75-100% EMB). These levels when compared to previous *C. elegans* embryonic lethality studies (28-51%) were higher [36,38-40], reflecting our ability to detect embryonic lethal phenotypes in a conserved set of genes using feeding RNAi techniques. We identified a smaller percentage of genes (21%; 7/34) that displayed a lower penetrance (defined as 10-75% EMB) of embryonic lethality, possibly reflecting functional redundancy or the potency of feeding RNAi for this set of genes. Overall, we identified a conserved class of genes from mammalian mitotic spindles necessary for embryonic development in *C. elegans*. 

### Characterization of 34 Identified embryonic lethal genes

**Figure 1:**
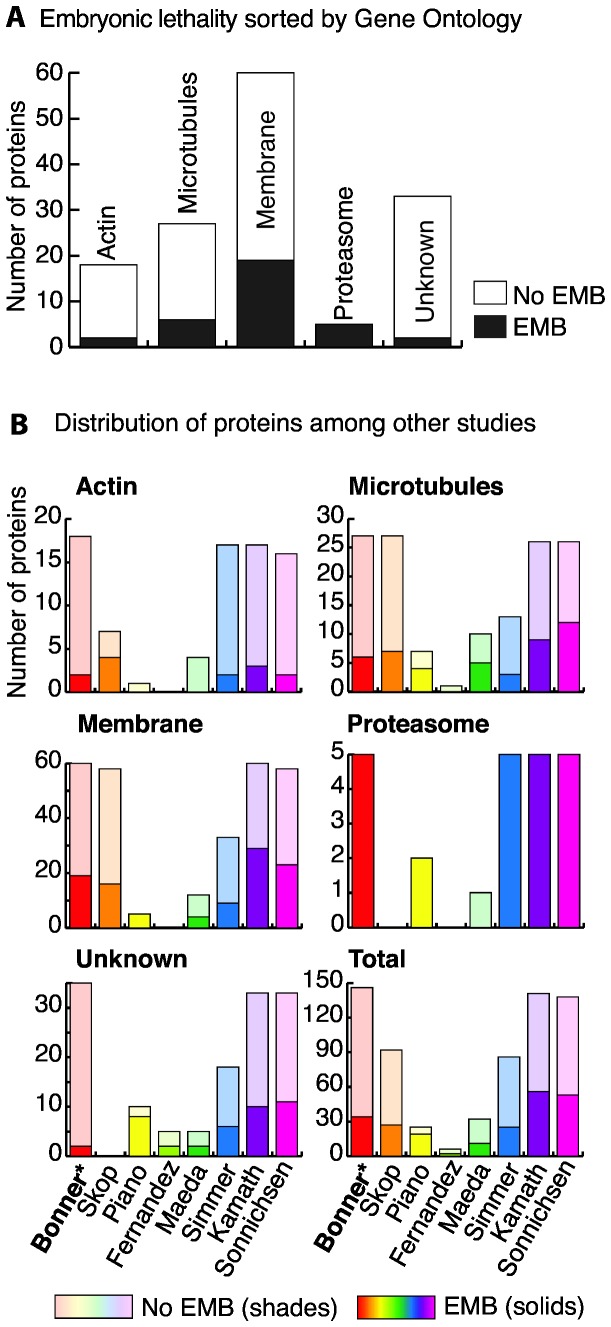
Summary of embryonic lethality data. A. Embryonic lethality sorted by Gene Ontology. Distribution of all genes sorted by Gene Ontology. Solid black bars indicate numbers of EMB genes, and white bars indicate numbers of No EMB genes. B. Distribution of proteins among other studies. All genes assayed in this study were checked for embryonic lethality status in the following previous studies: Skop et al., 2004, Piano et al., 2002, Fernandez et al., 2005, Maeda et al., 2001, Simmer et al., 2003, Kamath et al., 2003, and Sonnichsen et al., 2005. Solid colors indicate numbers of EMB genes, and shaded colors indicate numbers of No EMB genes. The Simmer, Kamath, and Bonner studies were performed by feeding RNAi, the Maeda study was performed by soaking RNAi, and the Piano, Skop, and Sonnichsen studies were performed by injection RNAi.

 To categorize this core set of embryonic lethal genes (n=34) by cellular location, we examined their distribution among GO categories (Figure 1A). Of the 34 embryonic lethal genes annotated, 56% (19/34 genes) belonged to the membrane group (Figure 1A). When we compared our GO data to phenotypes recorded for the same genes in previous screens (Figure 1B), we determined that the membrane group also was associated with the largest percentage of embryonic lethal genes in these screens (Skop et al., 2004, Simmer et al., 2003, Kamath et al., 2003, Sonnichsen et al., 2005), reinforcing the notion that membrane trafficking proteins are important and necessary cell division factors [15,34,36,40]. 

### Visual RNAi screen to identify genes involved in cell division

**Figure 2:**
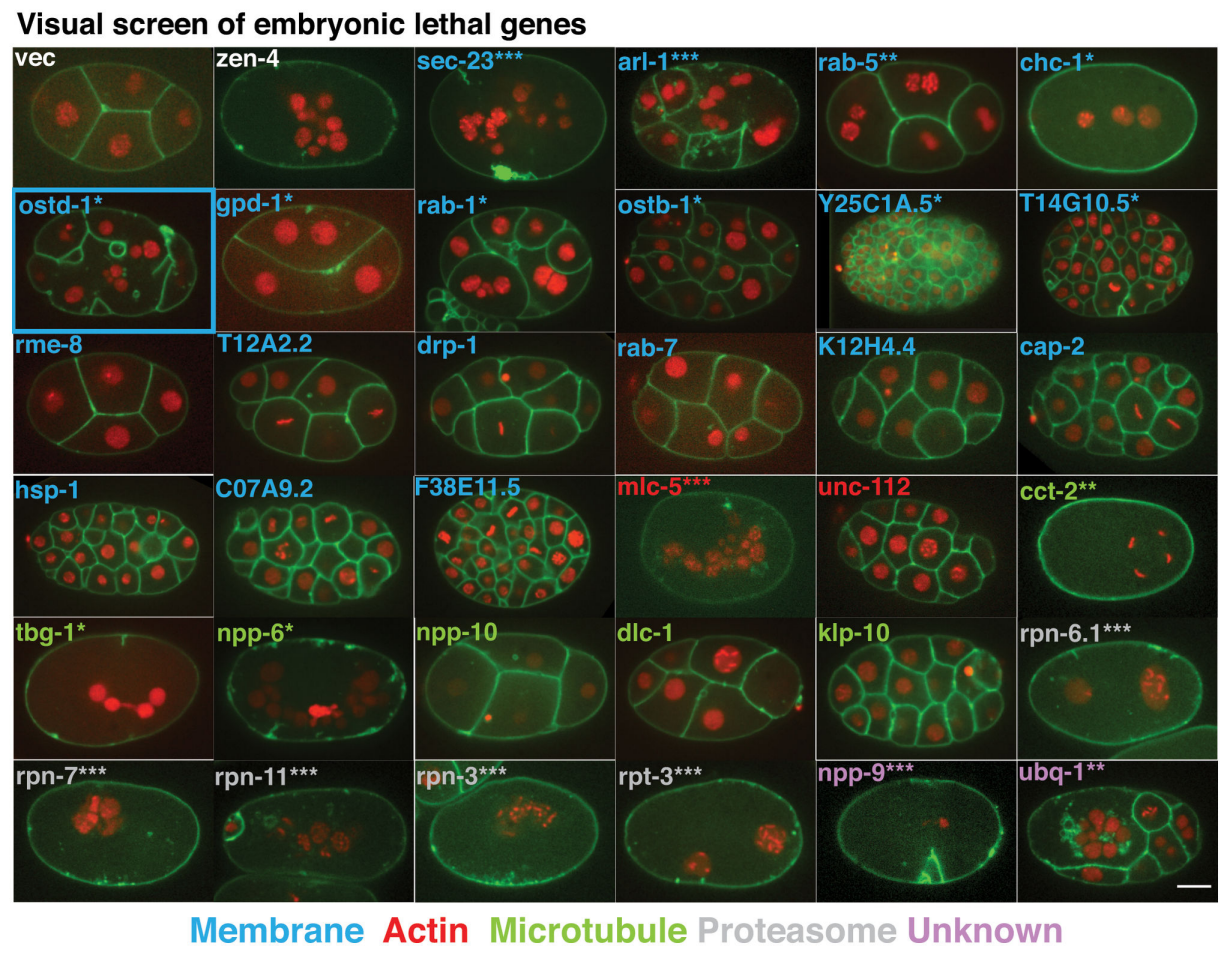
Visual screen of embryonic lethal genes. Confocal images were taken of embryos from a worm strain expressing GFP-PH ^PLC1delta1^; mCherry-HIS-58 after depleting the 34 embryonic lethal genes for 24 h. The feeding vector (vec) was our negative control, and zen-4 [101] was our positive control. The genes are ordered by GO term, shown by text color (blue for membrane, red for actin, green for microtubule, grey for proteasome, lilac for unknown). Within each GO term group, the genes are ordered by strength of phenotype: ***-Very Strong (75%+ multi-nucleate embryos), **-Strong (50% multi-nucleate embryos), *-Weak (25% multi-nucleate embryos), and Later phenotype (no multi-nucleate embryos). The visual screen identified twenty-one genes that were associated with multi-nucleate embryos, which indicates those genes are involved in cell division. The gene, *ostd-1* (outlined in blue), was chosen for further characterization in Figures 3-4. (Scale bar: 10 um).

To determine which of the 34 embryonic lethal genes played specific roles in cell division, a secondary visual RNAi screen was performed. Here, the presence of multi-nucleate embryos suggests that these genes are important for cell division. To identify candidates that were particularly important for cytokinesis, we used a *C. elegans* strain that labeled the plasma membrane (GFP-PH^PLC1delta1^) and the chromatin (mCherry-HIS-58). This allowed us to identify multi-nucleate embryos easily. Twenty-one (21) of the 34 embryonic lethal genes resulted in multi-nucleate phenotypes (Figure 2). Phenotypes varied from one-cell embryos with several nuclei (*mlc-5*, *rpn-11*, *cct-2*) to multi-cellular embryos with one or more cells with at least two nuclei (*ubq-1*, *ostd-1*, *arl-1*). We marked strength of phenotype for each gene in Figure 2. Very strong genes resulted in at least 75% multi-nucleate embryos (Figure 2,***), strong genes yielded 50% multi-nucleate embryos (Figure 2,**), and weak genes resulted in 25% multi-nucleate embryos (Figure 2,*). Genes not resulting in multi-nucleate embryos likely arrested from events later in development (*rme-8*, *unc-112*, cap *2*, K12H4.4). The largest group of genes (10/34) that was necessary for mitosis belonged to the membrane trafficking group (Figure 2, labeled in blue). Given the aberrant placement of cleavage furrows and cytokinesis failures in OSTD-1 RNAi-treated embryos, we sought to further characterize OSTD-1 in the early *C. elegans* embryo. 

### OSTD-1 is necessary for cell division

OSTD-1 is the ortholog of RPN2/Ribophorin II, which was identified from the published isolated mammalian mitotic spindle proteome from our laboratory [20]. RPN2 belongs to a conserved set of trans-membrane endoplasmic reticulum (ER) proteins called the OST complex. The OST complex is important for N-linked glycosylation [46-48], yet the functions of OST complex members outside of their roles in glycosylation have not been fully described. Mutations in RPN2 have been shown to belong to a group of inherited human disorders called, Congenital Disorders of Glycosylation (CDG)[49]. Silencing of human RPN2 also decreases drug resistant breast tumor and esophageal squamous cell carcinoma growth [50,51], indicating that RPN2 may function in cell division and be an important cancer therapeutic target. Lastly, in *C. elegans* and *Drosophila*, N-linked and O-linked glycosylation proteins, RIBO-1, SQV-1, SQV-2, Cog7 and Nessun Dorma, have been identified as being necessary for cell division events [46,52-58], suggesting that ER resident proteins play a necessary role in cell division. Given the importance of the OST complex in human disease and cell division defects we observed, we further investigated the role of OSTD-1 in mitosis.

**Figure 3:**
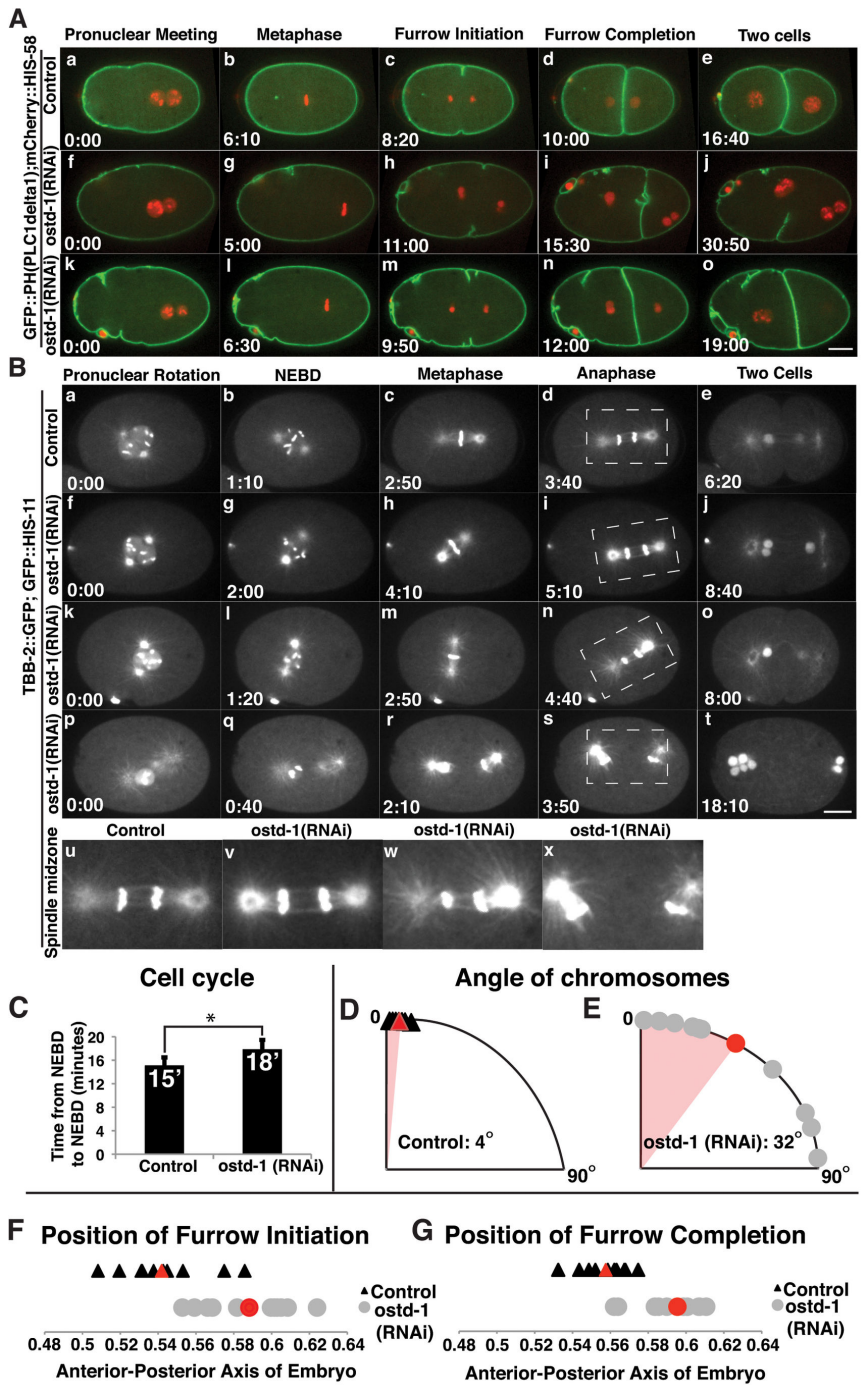
OSTD-1 depletion affects cell cycle time, cleavage furrow positioning, spindle alignment and completion of cytokinesis. **A**. **OSTD-1 depletions result in a cytokinesis defect**. Montages were made from time-lapse confocal movies of a worm strain expressing GFP-PH ^PLC1delta1^; mCherry-HIS-58. Images were selected from each movie to represent pronuclear meeting, metaphase, initiation of the cleavage furrow, completion of the cleavage furrow and two cells. In control embryos (Figure 3A, a-e), after the pronuclei meet, the mitotic spindle sets up at metaphase and the chromosomes segregate to opposite poles as the cleavage furrow ingresses and completes, resulting in two daughter cells. In the first *ostd-1* (RNAi) embryo (Figure 3A, f-j), the cleavage furrow ingresses but fails to complete, resulting in a multi-nucleate embryo (Figure 3A, j). In the second *ostd-1* (RNAi) embryo (Figure 3A, k-o), the mitotic spindle sets up in the posterior end of the embryo (Figure 3Al), indicating a spindle positioning defect, and cytokinesis completes successfully. All embryos were dissected in Shelton’s Growth Media. (Scale bar: 10 um). **B**. **OSTD-1 depletions result in spindle positioning and spindle midzone defects**. Montages were made from time-lapse confocal movies of a worm strain that expresses TBB-2-GFP and GFP-HIS-11 to visualize the mitotic spindle. Images were selected from each movie to represent pronuclear rotation, nuclear envelope breakdown (NEBD), metaphase, anaphase, and two cells. In the control embryo (Figure 3B, a-e), the pronuclei rotate and the nuclear envelope disassembles to allow the chromosomes to align at metaphase. The chromosomes align vertically as the mitotic spindle sets up along the anterior-posterior axis, and then the chromosomes segregate into two daughter cells. In the first *ostd-1* (RNAi) embryo (Figure 3B, f-j), the mitotic spindle assembles, but the chromosomes are tilted (Figure 3Bh), indicating a spindle-positioning defect. The second *ostd-1* (RNAi) embryo (Figure 3B, k-o) is another example of a mitotic spindle-positioning defect in that the chromosomes segregate vertically instead of horizontally (Figure 3B, m). The final *ostd-1* (RNAi) embryo (Figure 3B, p-t) displays a spindle midzone defect where the spindle midzone is completely absent (Figure 3B, r). A close-up image of each spindle midzone is shown in Figure 3B, (u-x). All embryos were dissected in Shelton’s Growth Media except for the embryo in 3B, (p-t), which was dissected in Egg Salts Buffer. The spindle midzone disruption phenotype was also observed in embryos dissected in Shelton’s Growth Media. (Scale bar: 10 um). **C**. **OSTD-1 depletions result in a significantly longer cell cycle time**. The cell cycle was quantified in control embryos and *ostd-1* (RNAi) embryos in a strain expressing GFP-PH ^PLC1delta1^; mCherry-HIS-58. The timing of the cell cycle was measured from the first nuclear envelope breakdown to the next nuclear envelope breakdown. Time is shown in minutes (n = 11 each, * indicates significance, p<0.005). **D**-**E**. **OSTD-1 depletions result in altered chromosome alignment at metaphase**. The angles of metaphase chromosomes were measured in control embryos and *ostd-1* (RNAi) embryos in a strain that expresses TBB-2-GFP and GFP-HIS-11. Angles were displayed on a 0-90 degree scatterplot graph where zero degrees equal a vertical alignment of chromosomes. The angles of the chromosomes in the control embryos were marked with triangles (D), and the red triangle and shading indicates the average of 4.2 degrees difference from vertical alignment. The angles of chromosomes in the *ostd-1* (RNAi) embryos (E) were marked with circles, and the red circle and shading indicates the average of 32.4 degrees difference from vertical alignment. (n = 10 each, p<0.05). **F**-**G**. **OSTD-1 depletions result in posterior shift of cleavage furrow ingression and completion**. We measured the furrow position in control and *ostd-1* (RNAi) embryos expressing GFP-PH ^PLC1delta1^; mCherry-HIS-58. The length of each embryo was set to 1 from the anterior pole to the posterior pole, and the relative positions of furrow initiation or furrow completion are marked along the anterior-posterior axis. The positions of each furrow initiation or completion in control embryos (F-G) are shown in scatterplots and are marked with triangles, with the average position marked with a red triangle. The positions of each furrow initiation or completion in *ostd-1* (RNAi) embryos (F-G) are marked with circles, with the red circle marking the average position. (n = 11 each, p<0.005).

To characterize the requirement of OSTD-1 in cell division, we depleted OSTD-1 using feeding RNAi and observed the early embryonic divisions using *in vivo* microscopy (Figure 3A, Movies S1-S3). We used a strain, GFP-PH^PLC1delta1^; mCherry-HIS-58, that marked the plasma membrane and the chromatin for these RNAi experiments. Depletion of OSTD-1 for 48h resulted in failures in both the initiation and the completion of cytokinesis in 25% of the embryos observed (n=4/17) (Figure 3A, f-j). Cytokinesis failures were also observed in the second and third cell divisions. OSTD-1 was identified in previous glycosylation screens as playing a role in cytokinesis in *C. elegans* [46,52], however several distinct phenotypes were not reported that we have identified. Here, we detected membrane aggregations near the anterior plasma membrane (n=16/17) (Figure 3A, f-j, 3A, k-o) and membrane protrusions along the cleavage furrow in *ostd-1* (RNAi) embryos (Figure 3A, i). Extra furrows appeared in 65% of *ostd-1* (RNAi) embryos (n=11/17), suggesting that the placement of the furrow is affected or a factor necessary for furrow placement is improperly targeted or regulated. In Figure 3A, (m-o), two cleavage furrows appear to ingress simultaneously from the top and bottom of the embryo, with one extra furrow lengthening even after cytokinesis completed. Cell cycle delays were also observed, consistent with previous results reported in Stevens and Spang, (2013) [52]. Here, depletion of OSTD-1 significantly slowed the cell cycle from an average of 15 minutes in control embryos to an average of 18 minutes in *ostd-1* (RNAi) embryos, measured from first nuclear envelope breakdown to the second nuclear envelope breakdown (Figure 3A,C). 

In *ostd-1* RNAi-treated embryos, the position of the cleavage furrow was placed significantly closer to the posterior end of the embryo (Figure 3A and 3F-G), which was not reported in previous *C. elegans* experiments investigating glycosylation proteins [46,52]. To investigate the altered furrow position defect further, we depleted OSTD-1 in a strain that expressed both TBB-2-GFP and GFP-HIS-11 to visualize the mitotic spindle and the position of the metaphase plate, respectively (Figure 3B, Movies S4-S7). Here, the spindle shifted toward the posterior end of the embryo at anaphase (Figure 3B, i). In addition, we measured the angle of the metaphase chromosomes just before anaphase (Figure 3B, 3D-E). In control embryos, the metaphase chromosomes averaged 4.2 degrees difference from vertical alignment, ranging from 1 to 8 degrees (Figure 3D). The angle of the chromosomes in *ostd-1* (RNAi) embryos averaged 32.4 degrees deviation from vertical, ranging from 1 to 86 degrees (Figure 3E), and two examples of this altered chromosome alignment are displayed in Figure 3B, f-o. OSTD-1 could play an indirect role in the trafficking of factors necessary for spindle alignment to the membrane. Some of our *ostd-1* RNAi-treated embryos also had disrupted spindle midzones, which has not been observed [46,52] (Figure 3B, p-t, u-x (shows a close up view);(n=3/12 embryos)). The tubulin-GFP signals at the centrosomes in *ostd-1* RNAi embryos (Figure 3B, x) were more intense than control embryos, suggesting that there may be fewer astral microtubules or altered microtubule dynamics in embryos where the spindle midzone is disrupted. Given the role of the OST complex and its localization in the ER membrane [46-48], we suggest that OSTD-1 may play an indirect role in microtubule dynamics by the lack of proper ER morphology within the spindle midzone and at centrosomes in *ostd-1* RNAi-treated embryos. 

### ER morphology defects are observed in *ostd-1* (RNAi) embryos

**Figure 4:**
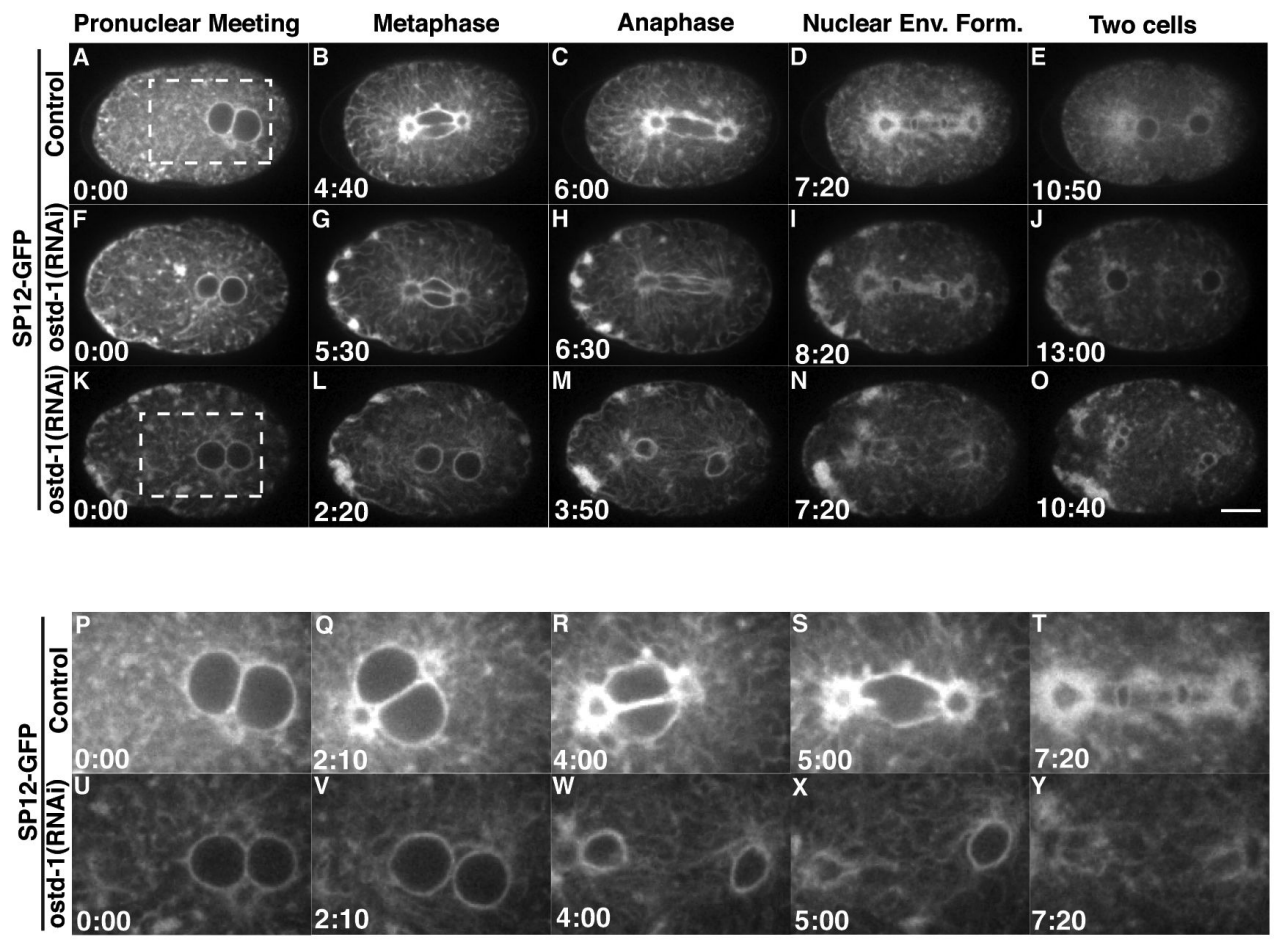
ER morphology is disrupted in *ostd-1* (RNAi) embryos. Montages were made from time-lapse confocal movies of a worm strain that expresses GFP-SP12 to visualize the endoplasmic reticulum (ER). Images were selected from each movie to represent pronuclear rotation, metaphase, anaphase, nuclear envelope formation, and two cells. (A) In the control embryo, the ER is spread as disperse sheets at pronuclear meeting. (B-C) During metaphase and anaphase, the ER surrounds the mitotic spindle in a reticular pattern. (D-E) At telophase, the nuclear envelope reassembles into one mononucleus in each daughter cell, and the ER disperses into sheets. (F-J) In the first *ostd-1* (RNAi) embryo, clumps of ER form near the plasma membrane in the anterior end of the embryo and persist through cell division. (F) At pronuclear meeting, patches of ER begin to aggregate near the plasma membrane at the anterior (n = 28/41). (G-H) The clumps of ER persist through metaphase and anaphase while the ER surrounds the spindle in a reticular pattern. (I) In telophase, the nuclear envelope reforms around the chromosomes. (J) The ER disperses into sheets into the cytoplasm. (K-O) In the second *ostd-1* (RNAi) embryo, the pronuclei meet but then segregate precociously before the mitotic spindle sets up (n = 1/41). (K) At pronuclear meeting, clumps of ER begin to aggregate in the anterior. (L-M) The pronuclei separate precociously as the ER becomes reticular. (N-O) The ER becomes more disperse and then two karyomeres form in both the AB and P1 cell. (P-T) A close-up view of the control embryo shows the ER surrounding the pronuclei in pronuclear meeting and rotation (P-Q). The ER surrounds the mitotic spindle during metaphase, anaphase and telophase (R-T). (U-Y) A close-up view of the second *ostd-1* (RNAi) embryo shows the pronuclei at pronuclear meeting and at the precocious separation (U-W). At the same times of metaphase and anaphase in the control embryo, the ER appears to surround the separated pronuclei instead of marking the outline of the mitotic spindle in the *ostd-1* (RNAi) embryo (W-X). During the same time of telophase, the ER disperses around the pronuclei. All embryos were dissected in Shelton’s Growth Media. (Scale bar: 10 um).

Since the homologs of OSTD-1 localize to the ER membrane, we wanted to determine if OSTD-1 plays a role in ER function during mitosis. We depleted OSTD-1 in a strain expressing an ER marker, SP12-GFP (Figure 4, Movies S8-S10) [59,60] to observe the dynamics of the ER. In the control embryo, the SP12-GFP marker revealed the ER as disperse sheets at pronuclear meeting (Figure 4A). During metaphase and anaphase, the ER surrounds the mitotic spindle in a reticular pattern (Figure 4B-C). At telophase, the nuclear envelope reassembles into one mononucleus in each daughter cell, and the ER disperses back into sheets (Figure 4D-E) [60,61]. In *ostd-1* (RNAi) embryos, most of the ER was disperse during pronuclear meeting, but ER aggregations began to occur near the anterior plasma membrane during pronuclear meeting (n=28/40) (Figure 4F). These ER aggregations or clumps persisted through mitosis as the rest of the ER surrounded the mitotic spindle in a reticular pattern in these RNAi-treated embryos (Figure 4F-I, L-O). The ER clumps persisted well into the next division even although most of the ER attempted to organize back into sheets (Figure 4J). Mild aggregates of ER form around the nucleus when RIBO-1 is knocked down, however this is distinct from the localization pattern in *ostd-1* RNAi-treated embryos [52]. The aggregations of ER in *ostd-1* RNAi-treated embryos are similar to mitotic ER clumps observed in *C. elegans* embryos with activated RAB-5 [61], except that the ER clumps in *ostd-1* RNAi-treated embryos formed in the anterior. OSTD-1 could play a role in maintaining ER morphology specifically in the anterior in some way. ER tubules often make contacts with the plasma membrane (called multiple contact sites or MCS) [62,63], and given that OSTD-1 is a trans-membrane ER protein, it could play a necessary role in maintaining the morphology of the ER through contacts with the plasma membrane [48]. 

By using the SP12-GFP marker we were also able to observe abnormal pronuclear movements in *ostd-1* RNAi-treated embryos (Figure 4K-O, U-Y). Here, pronuclear migration and meeting occurred normally, but the sperm and oocyte pronuclei separated precociously before the mitotic spindle set up (Figure 4K-M, U-X). In contrast to control embryos, where the pronuclear envelope broke down and the ER accumulated around the assembled mitotic spindle (Figure 4P-S), neither pronuclear envelope appeared to break down in this *ostd-1* RNAi embryo (Figure 4U-X). After the pronuclei separated precociously, multiple micronuclei formed in both the AB and P1 cell (Figure 4O) and cytokinesis ultimately failed when the cleavage furrow regressed. 

During the course of our experiments, multiple micronuclei or karyomeres were often observed in OSTD-1 depleted embryos. In *C. elegans*, other membrane-associated proteins, such as SYN-4, RAB-5 and RIBO-1 have been shown to be important for the formation of nuclei or karyomere fusion [52,61,64]. Karyomeres are small, round structures containing chromatin surrounded by a nuclear envelope and are an intermediate mitotic structure important for the formation of a nucleus at the end of mitosis. In 79% of the *ostd-1* (RNAi) two-celled embryos (n=11/14), small micronuclei were observed (Figure 3B j, t), instead of a single nucleus in the newly formed blastomeres, suggesting that the karyomeres failed to fuse. The ER (SP12-GFP) marker also localizes in aggregates around the karyomeres (Figure 4O). Similar karyomere fusion defects have been observed in *brambleberry* mutants in zebrafish [65,66]. Brambleberry is similar to Kar5p, a protein necessary for nuclear fusion in yeast [65]. Since the ER is contiguous with the nuclear membrane [67], we suspect that defects in ER morphology could affect the formation of nuclei in these embryos. Although the OST complex members play a predominant role in N-linked glycosylation, we have shown that OSTD-1 may play an additional role in maintaining ER morphology and dynamics during mitosis.

## Discussion

Our goal in this study was to identify important cell division factors from the mitotic spindle proteome through comparative genomics and two directed RNAi screens in *C. elegans*. We performed an embryonic lethality RNAi screen on 146 *C. elegans* orthologs of CHO mitotic spindle proteins [20] and identified 34 candidate genes that were then subjected to a secondary screen to detect multinucleate embryos. The visual screen yielded 21 genes that function in cell division. One candidate, OSTD-1, was chosen for further characterization based on the cell division defects observed. We found that depleting OSTD-1 predominantly leads to furrow placement defects, ER morphology dynamics alterations and cytokinesis failures. This strategy enabled us to identify and characterize new cell division factors and highlight the importance of ER proteins in mitosis.

Our identification of 21 cell division factors validates the power of directed screens. Combining a primary screen method to narrow down cell division candidates with a detailed visual assay produced an efficient, directed screen [68]. Candidates for our primary screen originated from the mitotic spindle proteome, increasing the likelihood that those proteins were factors in cell division. By definition, we examined conserved proteins, which are more likely to have essential functions in organisms, such as cell division [37,39]. Further, spindle candidates were filtered by GO categories relevant to cell division, likely leading to the high proportion of genes linked to multinucleate phenotypes. It is notable that over half of the embryonic lethal genes caused cytokinesis failures when they were depleted in the secondary assay. Previous directed screens also resulted in a higher percentage of candidates with phenotypes than whole genome-wide screens [15,34-36,38-40]. While large-scale screens are useful to obtain a global perspective on cellular functions, directed screens are likely to yield a deeper understanding of a particular process.

Our screen identified OSTD-1, a protein whose orthologs function in N-linked glycosylation in the ER [46]. If OSTD-1 plays a role in the canonical pathway of glycosylation, OSTD-1 could affect multiple cellular functions, since N-linked glycosylation modifies N-X-S/T sequences in hundreds of proteins [47,69]. To support this hypothesis, we observed multiple mitotic phenotypes when we depleted OSTD-1, including furrow placement defects, spindle alignment and spindle midzone disruptions, aberrant ER morphology and cytokinesis failures. Potential defects in glycosylation caused by depleting OSTD-1 may result in misfolded or mislocalized [70] cell division proteins. Because N-linked glycosylation affects proteins destined for the endomembrane system and endocytic proteins play an important role in cytokinesis, depleting OSTD-1 could result in the absence of certain membrane factors at the cleavage furrow. In animal cells, membrane trafficking and membrane maintenance at the furrow has been shown to be important for cytokinesis [71-76]. As *ostd-1* (RNAi) embryos display furrow defects and aggregations of membrane along the cleavage furrow, OSTD-1 could play a role in glycosylation of proteins or trafficking of membrane factors necessary for these events. In plants and *S. pombe*, proteins involved in glycosylation have been shown to be necessary in the formation of new cell walls or septa [77,78], suggesting that OST complex members have a conserved role in cell division and the trafficking of membrane to the new cell wall or to the cleavage site. Future work will reveal if OSTD-1 functions in glycosylation and the importance of glycosylation in cell division.

The defects in ER morphology we observed could also indicate that OSTD-1 functions in the maintenance and dynamics of the ER structure during the cell cycle. Large aggregations of ER accumulated near the anterior plasma membrane when OSTD-1 was depleted, suggesting that OSTD-1 is necessary for the proper distribution of ER during embryonic development. Whether these aggregations are ER cisternae or not is unclear. Similar ER morphology defects have been observed in *rab-5*, *rab-11*, car-*1*, and *zyg-8* RNAi-treated embryos demonstrating the importance of ER morphology during cell division [59,61,79,80]. ER morphology was described as being mildly affected in *ribo-1* RNAi-treated embryos [52], suggesting that OSTD-1 may play a distinct role in maintaining ER morphology. During mitosis, members of the OST complex could play additional roles in maintaining ER morphology aside from a role in glycosylation. RIBO-1, for example, has been shown to play a role in chromosome segregation [52]. The polarized ER aggregations observed in OSTD-1 depletions are also distinct from the distribution of ER aggregates reported in *ribo-1*, car-*1* and *lpin-1* RNAi-treated embryos or the more disperse distribution of ER in *rab-5* and *yop-1/ret-1* depletions [59,61,81], suggesting that OSTD-1 may be involved in maintaining ER morphology specifically in the anterior. It is not clear how the polarization of ER functions during mitosis, but transitional ER (tER) sites have been shown to be polarized in the hyphal tips of fungi where growth and morphogenesis occur [82]. Lastly, given the transmembrane structure of the OSTD-1 protein and close association with other ER proteins that mediate plasma membrane contacts, depletion of OSTD-1 could disrupt ER and plasma membrane contacts sites (or MCSs) [62,63]. In yeast, loss of the proteins necessary to maintain the MCSs (called VAP proteins; Scs2 and SCS22) results in ER collapse [83-85]. OSTD-1 could function in maintaining these ER-plasma membrane contacts. Although the associations with the VAP proteins are speculative, the ER clumping or ER collapse phenotypes we observe in *ostd-1* RNAi embryos hint that OSTD-1 could function in maintaining proper ER-plasma membrane contacts. Investigating the ultrastructure of the ER and plasma membrane in *ostd-1* RNAi-treated embryos is necessary to determine if ER-plasma membrane contacts are important during mitosis.

The role of OSTD-1 in furrow positioning could indicate that there is a link between the ER and membrane trafficking machinery or plasma membrane during mitosis. In COS7 cells, the ER has been shown to be tightly associated with endosomes and this association is maintained over time independent of microtubules [86]. In *S. cerevisiae*, the ER is often associated with sites in the plasma membrane (MCS) [62,63]. Trans-membrane proteins, such as Ist2, tether the ER membrane to the plasma membrane and deletions of these ER tether proteins result in a collapse of cortical ER away from the periphery of the cell [84], similar to what we have observed with *ostd-1* fRNAi embryos (Figure 4). The collapse of ER in *ostd-1* fRNAi embryos may influence the progress of cell division. Cortical ER has been shown to restrict the localization of Mid1p, a protein that positions the acto-myosin ring in *S. pombe* [87], suggesting that cortical ER and plasma membrane connections likely maintain the organization of the ER and localization of cytokinesis factors. The disrupted ER we observe in OSTD-1 RNAi-treated embryos could lead to the dispersal of factors necessary to assemble the acto-myosin ring, given that we observe that 65% of our *ostd-1* fRNAi embryos failed to assemble furrows in the proper place. Proper ER localization and morphology has been shown to be important in axons for localized translation of signals at synapses and during polarized cell growth [88,89], suggesting that proper ER structure and possibly ER-plasma membrane contacts could be important for the assembly of the machinery necessary for cell division. 

To determine if other ER resident proteins have been shown to be important for cytokinesis, we found that in *C. elegans*, only trans-membrane localized ER proteins are important during cytokinesis [15,90,91]. A lumenal ER protein, ENPL-1 (ortholog of endoplasmin/GRP94) functions only in chromosome segregation, whereas the trans-membrane localized ER proteins, CNX-1 (calnexin homolog) and SEC 31, when depleted result in cytokinesis defects [15]. This suggests that trans-membrane ER proteins, like OSTD-1, could function in the overall integrity of the ER structure and dynamics during mitosis, such that when these proteins are depleted failures in cytokinesis occur. Proper ER structure during mitosis could play a necessary role in compartmentalizing the signals or machinery important for cell division, but future work is needed to determine this.

Probing the role of OSTD-1 in cell division raises questions about the importance of ER proteins in cell division. The connection between the ER and cell division has been uncovered over the years, with reports of ER proteins performing essential roles during cell division [46,53-55,92], yet very little is know about their distinct roles. Recently, a newly uncharacterized ER protein, REEP3/4, has been shown to maintain proper ER morphology and chromosome segregation during mitosis [93], supporting our findings that ER protein function and ER morphology are important during mitosis. This data and our work presented here suggest that ER proteins likely play multiple roles in cell division aside from their functions in the ER. OSTD-1 joins this growing group of ER proteins that have been implicated in cell division [15,59,79,93], reflecting an important role for ER proteins and ER morphology during mitosis. Further research will reveal the importance of maintaining proper ER morphology and dynamics during mitosis.

## Materials and Methods

### Worm Strains

The following strains were used: N2 wild type [94], OD95 (GFP-PH^PLC1delta1^;mCherry-HIS-58)[95], TY3558 (TBB-2-GFP;GFP-HIS-11)[15] and WH327 (SP12-GFP)[60]. The wild type worm strain, N2, was maintained as described in Brenner [94]. Transgenic worm strains (Green fluorescent protein and mCherry) were cultured at 20°C (OD95, TY3558 and WH327). 

### RNA Interference

RNA interference was delivered by the feeding method [96]. All bacterial strains used, except for the GFP feeding clone, were obtained from the Ahringer feeding library [36]. The GFP feeding clone (pSG006) was a kind gift from Scott Kennedy. Bacterial cultures were incubated for 16 h overnight. Feeding RNAi plates were seeded with 100 ul of culture and dried for 24 h at room temperature while covered. 

### Embryonic Lethality Screen

The embryonic lethality screen was performed on N2 worms at 20°C. Feeding RNAi plates were poured, dried on the bench top in the dark for 4-5 days, and then seeded with 100 ul bacterial culture from the Ahringer feeding library [36]. The feeding vector, PL4440 alone, and the GFP feeding plasmid (pSG006), were used for negative controls, and the *dyn-1* and *zen-4* fRNAi strains were used for positive controls. In quadruplet, 1 L4 N2 worm was placed on an fRNAi plate for 24 h and then moved to another plate seeded with the same bacteria for 24 h. Hatched worms and embryos were counted 18-24 h after removal of the mother. Genes corresponding to more than 10% embryonic lethality were considered embryonic lethal genes. Genes resulting in 75-100% embryonic lethality were categorized as “High EMB,” and genes resulting in 10-74.99% embryonic lethality were categorized as “Low EMB.”

### Secondary Screen

The secondary screen was performed on OD95 worms at 20°C. OD95 were placed on plates seeded with selected positive candidates or controls for 24 h. Embryos were dissected from OD95 adult worms in Egg Salts buffer in watch glasses and deposited on 2% agar pads on slides. Coverslips were placed on the agar pads and sealed to the slides with Vaseline. Four representative embryos from each candidate or control were selected and imaged. If multiple nuclei were observed in three or four embryos, that candidate was noted as having a “very strong” phenotype, if multiple nuclei were seen in two out of the four embryos, that gene was noted as having a “strong” phenotype, and if multinucleation was observed in only one embryo, that candidate was designated as having a “weak” phenotype. Candidates that were associated with no multi-nucleation were categorized as later embryonic defects. Exposure times for OD95 images in the multinucleate embryo screen were either 500ms for GFP-PH ^PLC1delta1^ and 750 ms for mCherry-HIS-58 or 100 ms for GFP-PH ^PLC1delta1^ and 500 ms for mCherry-HIS-58.

### Imaging

Images were captured on a 200M inverted, Axioscope microscope (Carl Zeiss) equipped with a spinning disk confocal scan head (QLC100; VisiTech, Sunderland, United Kingdom). For the multi-nucleate embryo screen, the ORCA ER camera (Hamamatsu) was operated through OpenLab software (Improvision, Coventry, United Kingdom) and MetaMorph software (Molecular Devices, LLC, Sunnyvale, CA, USA). For the detailed characterization of OSTD-1, the ORCA ER camera (Hamamatsu) was operated through MetaMorph version 7.7.7.0 (Molecular Devices, LLC, Sunnyvale, CA, USA). Images were taken every 10 s for all movies. ImageJ software [97] was used for rotating and cropping TIFF files for movies and figures.

### OSTD-1

To investigate the role of OSTD-1 in cell division, time-lapse movies were recorded at 10 s intervals. OD95, TY3558 and WH327 worms were subjected to 48 h feeding time to deplete *ostd-1*. Embryos were dissected in Shelton’s Growth Media, except for one TY3558 embryo that was dissected in Egg Salts buffer (Figure 3p-t), and all were mounted on 2% agar pads [98,99]. Coverslips were placed on the agar pads and sealed to the slides with Vaseline. Cell cycle time was quantified by measuring 11 movies each for OD95 control experiments and OD95 OSTD-1 depletions. Cell cycle time was calculated from the first nuclear envelope breakdown to the next nuclear envelope breakdown. The positions of the cleavage furrow initiation and completion were calculated from the same set of 22 movies. Each embryo was made a relative length of 1 along the anterior-posterior axis, and the position of initiation and completion were measured on the anterior-posterior axis. For chromosome alignment experiments, we used TY3558 to image the mitotic spindle. Exposure time for TY3558 was 250 ms for TBB-2-GFP and GFP-HIS-11. The chromosome alignment was quantified by analyzing 10 movies each for TY3558 control and TY3558 *ostd-1* experiments. The angle of the chromosomes at the last image of metaphase was measured with respect to a vertical line in the embryo. Zero degrees equaled a vertical line, and the angles recorded represented deviation of the chromosome angle from vertical. We used WH327 to image the ER. Exposure time for WH327 was 500 ms or 75 ms for SP12-GFP.

### Bioinformatics

CHO candidate proteins included proteins from the CHO mitotic spindle proteome associated with actin, microtubule, membrane and proteasome GO terms and proteins not associated with GO terms (unknown) [20]. In order to select *C. elegans* orthologs of CHO candidates, we performed reciprocal BLAST [http://www.ncbi.nlm.nih.gov] on each CHO candidate, checked TreeFam [www.treefam.org] for each ortholog in established phylogenetic trees and evaluated the similarity in protein domains in Pfam [http://pfam.sanger.ac.uk/]. Next, we confirmed each determination of corresponding orthologs on the following databases, Uniprot [100], InterPro [http://www.ebi.ac.uk/interpro/] and Ensembl [www.ensembl.org]. After orthologs were selected, a PubMed literature search was conducted [www.ncbi.nlm.nih.gov/pubmed/] to eliminate orthologs with characterized roles in mitosis and cytokinesis (n=33). All orthologs were checked for inducing embryonic lethality, sterility, and mitosis, meiosis or cytokinesis defects in the following previous studies: Kamath et al., 2003, Simmer et al., 2003, Fraser et al., 2003, Sonnichsen et al., 2005, Maeda et al., 2001, Skop et al., 2004, Piano et al., 2002, Gonczy et al., 2000, and Fernandez et al., 2005. Genes resulting in embryonic lethality in any of the previous screens were considered “EMB.” Genes resulting in no embryonic lethality in any of the previous screens were considered “No EMB.”

In order to determine whether a protein was associated with human disease, mammalian orthologs were assessed for gene phenotype relationships on the Online Mendelian Inheritance in Man (OMIM) website (Online Mendelian Inheritance in Man, OMIM. McKusick-Nathans Institute of Genetic Medicine, Johns Hopkins University (Baltimore, MD), {December 2012}. http:://omim.org/). Orthologs with one or more gene phenotype relationship were recorded as positive for an association with human disease.

## Supporting Information

Movie S1 **Movie for Figure 3A, a-e, cropped time series.** Example of control embryo expressing GFP-PH^PLC1delta1^; mCherry-HIS-58.
(MOV)

Movie S2 **Movie for Figure 3A, f-j, cropped time series.** Example of ostd-1 RNAi-treated embryo expressing GFP-PH^PLC1delta1^; mCherry-HIS-58, with cytokinesis failure.
(MOV)

Movie S3 **Movie for Figure 3A, k-o, cropped time series.** Example of ostd-1 RNAi-treated embryo expressing GFP-PH^PLC1delta1^; mCherry-HIS-58, with extra cleavage furrow.
(MOV)

Movie S4 **Movie for Figure 3B, a-e, cropped time series.** Example of control embryo expressing TBB-2-GFP; GFP-HIS-11.
(MOV)

Movie S5 **Movie for Figure 3B, f-j, cropped time series.** Example of ostd-1 RNAi-treated embryo expressing TBB-2-GFP; GFP-HIS-11, with altered spindle orientation.
(MOV)

Movie S6 **Movie for Figure 3B, k-o, cropped time series.** Example of ostd-1 RNAi-treated embryo expressing TBB-2-GFP; GFP-HIS-11, with altered spindle orientation.
(MOV)

Movie S7 **Movie for Figure 3B, p-t, cropped time series.** Example of ostd-1 RNAi-treated embryo expressing TBB-2-GFP; GFP-HIS-11, with spindle midzone disruption.
(MOV)

Movie S8 **Movie for Figure 4A-E, cropped time series.** Example of control embryo expressing GFP-SP12.
(MOV)

Movie S9 **Movie for Figure 4F-J, cropped time series.** Example of ostd-1 RNAi-treated embryo expressing GFP-SP12, with anterior aggregates of ER.
(MOV)

Movie S10 **Movie for Figure 4K-O, cropped time series.** Example of ostd-1 RNAi-treated embryo expressing GFP-SP12, with precocious separation of pronuclei.
(MOV)

Table S1 **This table includes the embryonic lethality data from the *C. elegans* orthologs of the mammalian candidate genes identified in (Bonner et al., 2011).** Section one includes the 146 *C. elegans* genes assayed for embryonic lethality. Section two includes 34 genes not assayed because they have been previously characterized in the literature. Section three includes 66 genes not assayed because they were not in the feeding RNAi library.
(XLS)
