# Correction: Profiling of the Mammalian Mitotic Spindle Proteome Reveals an ER Protein, OSTD-1, as Being Necessary for Cell Division and ER Morphology

**DOI:** 10.1371/journal.pone.0171399

**Published:** 2017-01-30

**Authors:** Mary Kate Bonner, Bo Hwa Han, Ahna R. Skop

The third author’s middle initial is missing. The correct name is: Ahna R. Skop. The correct citation is: Bonner MK, Han BH, Skop AR (2013) Profiling of the Mammalian Mitotic Spindle Proteome Reveals an ER Protein, OSTD-1, as Being Necessary for Cell Division and ER Morphology. PLoS ONE 8(10): e77051. doi:10.1371/journal.pone.0077051
